# Multimodal approaches in female healthcare: the role of integrative bioinformatics

**DOI:** 10.1186/1878-5085-5-S1-A60

**Published:** 2014-02-11

**Authors:** Olga Golubnitschaja

**Affiliations:** 1 Molecular Diagnostics, Radiological Clinic, Medical Faculty, Friedrich-Wilhelms-University of Bonn, Germany

## 

Due to the current demographic trends in favour of elderly populations, more complex clinical situations such as co-development of severe disorders and co-morbidities should be considered as the persistent challenge that requires new strategies in healthcare aiming at both better life-quality of corresponding patient cohorts and improved economy of healthcare.

## 

**Figure 1:**
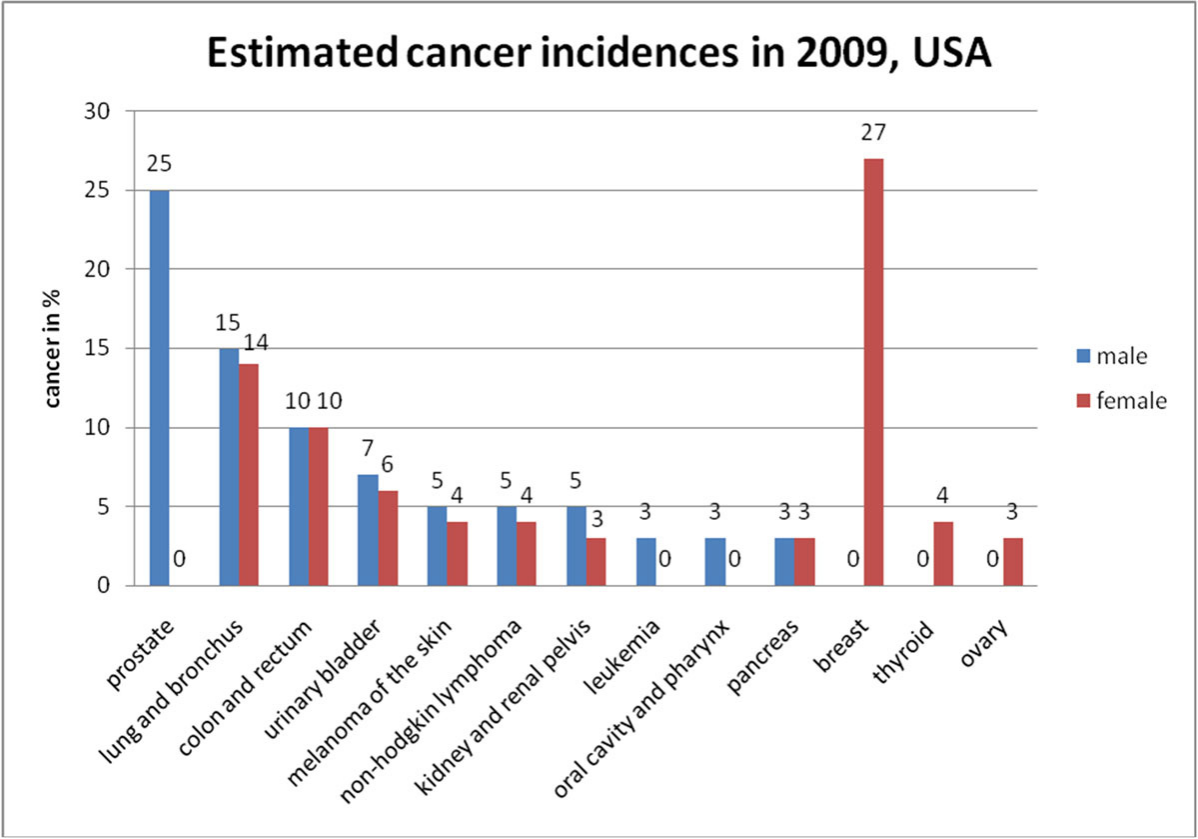
Cancer incidence as recorded in the USA, 2009 [1].

**Figure 2:**
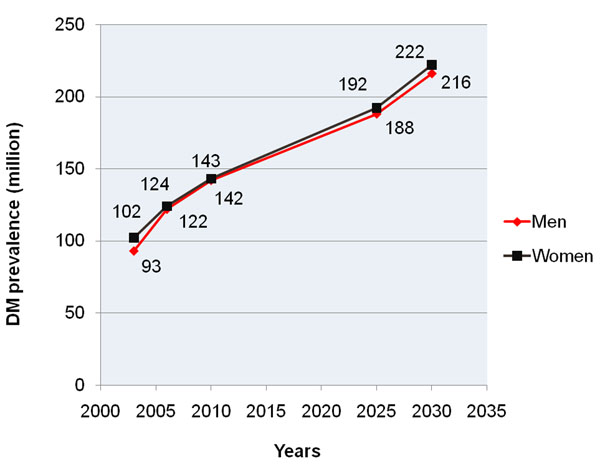
Global prevalence of diabetes type 2 in men and women as recorded and prognosed for the years 2002-2030 [2]

The most frequent female pathologies are the cardiovascular disease, breast cancer and Diabetes Mellitus type 2 – see figures 1 and 2. Further, the risk factors moderating the individual outcomes have been demonstrated to be common for the most frequent pathologies in females: progressive aging, overweight, poor diet, physical inactivity, chronic inflammation and depression. Modifiable risk factors persist from childhood and adolescence into adulthood and tend to cluster with synergistic negative effects for consequent manifestation of co-morbid pathologies [3].

Integrative bio-informatics is the key instrument to analyse the common risk factors as well as their individual and synergistic effects. Frequent *versus* rare co-morbidity profiles in patient cohorts should be created for the effective prediction, targeted prevention and advanced treatment regiments tailored to the patient. To implement this, corresponding pilot studies should be designed aiming in

1. creation of predictive molecular profiles of the disease, co-morbidities and pre-stages;

2. understanding of disease initiation: molecular, sub-cellular, cellular, inter-cellular, and single-organ levels as well as the organism as the whole;

3. disease progression should be considered as an interplay between promoters, contributors and inhibitors resulting in individual outcomes (similar to a geometric system of vectors by summarizing directions and contributing power);

4. tailored treatment algorithms: specific targets, targeted pathways, multi-drug cocktails, frequent *versus* rare complications and co-morbidities, multi-modal approaches;

5. effective primary and secondary prevention of co-morbidities but not single diseases;

6. new strategies in overall psychological supervision of individuals at high risk and patients at different disease stages;

7. new European and inter/national guidelines for population screening that would make good use of gathered knowledge to advance cost-effective healthcare and improve individual outcomes;

8. promotion of integrative (bio)medicine to force practice-oriented research and cost-effective healthcare;

9. creation of a critical mass of experts stuffing for integrative (bio)medical disciplines;

10. education at two levels, namely in professional groups and in general population.

For this integrative proposal following professional groups are essential: bio-informaticians, researchers with the relevant expertise, healthcare providers, relevant industrial partners, educators, collaborating patient groups, policy-makers, funding bodies.
